# Maternal Overweight and Obesity Alter Neurodevelopmental Trajectories During the First Year of Life: Findings from the OBESO Cohort

**DOI:** 10.3390/children12101385

**Published:** 2025-10-14

**Authors:** Arturo Alejandro Canul-Euan, Jonatan Alejandro Mendoza-Ortega, Juan Mario Solis-Paredes, Héctor Borboa-Olivares, Sandra Martínez-Medina, Carmen Hernández-Chávez, Gabriela Gil-Martínez, Erika Osorio-Valencia, Mariana Torres-Calapiz, Blanca Vianey Suárez-Rico, Isabel González-Ludlow, Carolina Rodríguez-Hernández, Ameyalli Rodríguez-Cano, Enrique Reyes-Muñoz, Ignacio Camacho-Arroyo, Sonia L. Hernandez, Otilia Perichart-Perera, Guadalupe Estrada-Gutierrez

**Affiliations:** 1 Department of Immunobiochemistry, Instituto Nacional de Perinatología, Mexico City 11000, Mexico; dr.canul.neurodesarrollo@gmail.com (A.A.C.-E.); jonatan.mdz93@gmail.com (J.A.M.-O.); 2 Centro de Estudios Avanzados sobre Violencia-Prevención (CEAVI-P), Instituto Nacional de Pediatría, Mexico City 04530, Mexico; 3 Departamento de Inmunología, Escuela Nacional de Ciencias Biológicas, Instituto Politécnico Nacional, Mexico City 11000, Mexico; 4 Department of Reproductive and Perinatal Health Research, Instituto Nacional de Perinatología Isidro Espinosa de los Reyes, Mexico City 11000, Mexico; juan.solis@inper.gob.mx; 5 Community Interventions Research Branch, Instituto Nacional de Perinatología Isidro Espinosa de los Reyes, Mexico City 11000, Mexico; hector.borboa@inper.gob.mx (H.B.-O.); blancasuarezrico@gmail.com (B.V.S.-R.); 6 Department of Developmental Neurobiology, Instituto Nacional de Perinatología Isidro Espinosa de los Reyes, Mexico City 11000, Mexico; sandra.martinez@inper.gob.mx (S.M.-M.); karmenhch1966@gmail.com (C.H.-C.); geemege2001@yahoo.com.mx (G.G.-M.); erika.osorio@inper.gob.mx (E.O.-V.); mariana.torres@inper.gob.mx (M.T.-C.); 7 Nutrition and Bioprogramming Coordination, Instituto Nacional de Perinatología Isidro Espinosa de los Reyes, Mexico City 11000, Mexico; tmm.isabel.gonzalez@gmail.com (I.G.-L.); carolina.rdz94@gmail.com (C.R.-H.); rocameyalli@gmail.com (A.R.-C.); 8 Coordination of Gynecological and Perinatal Endocrinology, Instituto Nacional de Perinatología Isidro Espinosa de los Reyes, Mexico City 11000, Mexico; enriquereyes@inper.gob.mx; 9 Unidad de Investigación en Reproducción Humana, Instituto Nacional de Perinatología-Facultad de Química, Universidad Nacional Autónoma de México, Mexico City 11000, Mexico; ignacio.camacho@inper.gob.mx; 10 Surgery Department, Section of Pediatric Surgery, The University of Chicago, Chicago, IL 60637, USA; soniah@uchicago.edu

**Keywords:** developmental programming, infant, brain development, the first 1000 days, pregestational body mass index, pregnancy

## Abstract

**Background/Objectives:** Overweight and obesity during pregnancy are metabolic risk factors that may compromise offspring brain development. The first 1000 days of life represent a critical window in which neurodevelopmental trajectories are shaped by intrauterine and early-life exposures. The 6- and 12-month milestones are key checkpoints where deviations may emerge, and interventions are most effective. This study evaluated the association between maternal pregestational weight status and infant neurodevelopment at 6 and 12 months of age. **Methods:** Mother and infant pairs from the OBESO perinatal cohort in Mexico City were included. Women in the first trimester of pregnancy were classified as normal weight and overweight/obesity according to their pregestational body mass index (pBMI), calculated from self-reported pre-pregnancy weight. Infant neurodevelopment was assessed at 6 and 12 months using the Bayley Scales of Infant Development III, Third Edition (BSID-III). Descriptive, bivariate and multiple linear regression analyses with mixed effects correction were conducted. **Results:** Among 97 mother–infant pairs, infants of mothers with overweight/obesity had lower language and socio-emotional scores at 12 months. Higher maternal pBMI was correlated with lower motor scores at 6 and 12 months, and with lower language scores at 12 months. Longitudinal analysis showed that maternal overweight/obesity was associated with a significant decline in language development from 6 to 12 months. (*p* = 0.002). **Conclusions:** Maternal pregestational overweight or obesity may negatively influence early neurodevelopment, particularly affecting language and cognitive domains during the first year of life. These early deficits could reflect alterations in intrauterine programming associated with maternal metabolic status.

## 1. Introduction

The global prevalence of overweight and obesity has dramatically risen, particularly among women of reproductive age [1]. In Mexico, 68% of women of childbearing age are affected by overweight or obesity, according to the 2022 National Health and Nutrition Survey [2]. These metabolic conditions during pregnancy can have detrimental effects of fetal development, including neurodevelopmental outcomes [3,4]. Emerging evidence suggests that maternal pregestational body mass index (pBMI) plays a crucial role in shaping early-life brain development, potentially altering trajectories that extend into childhood and adolescence [5,6,7,8,9,10,11].

Pregestational overweight and obesity are associated with a range of adverse outcomes, including altered micronutrient transfer, inflammatory processes, oxidative stress, and lipotoxicity, which may disrupt fetal brain development [12,13]. These conditions may also influence infant outcomes through postnatal factors such as altered breastmilk composition and maternal–infant interaction [14]. Epigenetic modifications and changes in the infant gut microbiome linked to maternal metabolic status further support the concept of developmental programming [3].

Neurodevelopmental disorders affect approximately 10–20% of the global population [15] and share common pathophysiological mechanisms, many of them may originate even before conception [16,17]. In this context, the first 1000 days of life—from conception to two years of life—constitute a biologically sensitive window in which developmental trajectories are established. [18]. During this time, the brain undergoes rapid structural and functional changes and is particularly vulnerable to nutritional and environmental insults [15,19]. Each month contributes to a dynamic path shaped by genetic, epigenetic, and environmental influences. Within this continuum, the 6- and 12-month marks represent critical checkpoints: at 6 months, the introduction of complementary feeding and the emergence of early motor and social milestones provide opportunities for monitoring divergence; at 12 months, language, mobility, and autonomy accelerate, making atypical trajectories more visible [16,20,21]. These moments are not only clinically relevant but also strategic for parental education, policy engagement, and early intervention. In this sense, the first 1000 days are foundational, and the 6- and 12-month nodes are pivotal points where trajectories can be reinforced, redirected, or repaired [21,22]. While numerous studies have examined associations between maternal obesity and offspring neurodevelopment, most rely on cross-sectional assessments and lack data on early developmental trajectories.

Longitudinal assessments provide deeper insight into developmental patterns and the timing of risk. Previous studies, such as those in the PREOBE and PREDO cohorts, have linked maternal obesity with lower cognitive and motor outcomes in early childhood [23,24]. Recently, maternal overweight has been identified as a risk factor that predicts neurodevelopmental transition patterns toward lower scores from the infant to early childhood stage [25]. However, limited evidence exists regarding how maternal pBMI may influence neurodevelopment as early as 6 and 12 months. This study aimed to fill this gap by evaluating the association between maternal weight status and neurodevelopmental trajectories during the first year of life using data from the OBESO perinatal cohort.

## 2. Materials and Methods

### 2.1. Study Population

This study is part of the OBESO (Epigenetic and Biochemical Origin of Overweight and Obesity), prospective perinatal cohort conducted at the National Institute of Perinatology (INPer) in Mexico City. The OBESO study investigates how maternal nutrition, lifestyle, and metabolic and inflammatory profiles influence offspring neurodevelopment and body composition. The study protocol was approved by the INPer IRB (Register No. 3300-11402-01-575-17; 14 January 2024, 11 January 2021), and all participants provided written informed consent.

The study enrolled 238 mother-infant pairs as part of the ongoing OBESO perinatal cohort. Pregnant women were recruited during their first prenatal visit (11–14 weeks of gestation) at the Department of Maternal-Fetal Medicine (January 2017–January 2020). The inclusion criteria required mothers to be at least 18 years old at enrollment, carrying a singleton pregnancy, and free from preexisting chronic diseases (type 2 diabetes, hypertension, thyroid dysfunction, autoimmune, cardiac, hepatic, or renal disorders) or medication use affecting metabolism (e.g., insulin, corticosteroids, metformin). Mothers were excluded if they used tobacco or recreational drugs, had a clinical diagnosis of fetal abnormalities, or reported any medications that could affect their physical or mental health. Newborn without complete neurodevelopment assessments at 6 and 12 months were eliminated from this study.

### 2.2. Maternal and Infant Variables

Maternal pregestational weight was self-reported, and height was measured using the Lohman’s technique [26] with a digital stadiometer (SECA 246; SECA GmbH & Co. KG, Hamburg, Germany). pBMI was calculated and classified using WHO criteria [27]: normal weight (18.5−24.99 kg/m^2^) and overweight/obesity (≥25 kg/m^2^). Maternal intelligence quotient (IQ) was assessed using the Wechsler Abbreviated Scale of Intelligence (WASI) [28]. Additional variables included maternal age, parity, educational attainment, and gestational weight gain (GWG) were categorized as insufficient, adequate, or excessive according to the Institute of Medicine guidelines [29].

Obstetric complications such as gestational diabetes (GDM), gestational hypertension, preeclampsia, and fetal growth restriction were recorded using standard clinical definitions [30,31]. Gestational age at birth was estimated based on the ultrasound in the first trimester of pregnancy and corrected gestational age was used for preterm infants. Mode of delivery, preterm birth (<37 weeks) [32], Apgar scores, and neonatal anthropometric outcomes (birth weight, length, head circumference) [26] were also documented. Newborn BMI (nBMI) was calculated. Trained dietitians performed all neonatal measurements within 48–72 h of delivery using calibrated equipment (Tanita WB-3000; Tanita Corporation, Tokyo, Japan);infantometer (SECA model 207; SECA GmbH & Co. KG, Hamburg, Germany); and measuring tape (SECA model 212; SECA GmbH & Co. KG, Hamburg, Germany).

### 2.3. Neurodevelopment Assessment

Infant neurodevelopment was evaluated at 6 and 12 months of age using the Bayley Scales of Infant Development, Third Edition (BSID-III). This standardized tool assesses four domains: cognitive, language, motor, and socio-emotional development [33]. Tests were administered by trained child psychologists at the research facility. Each assessment session lasted 30–40 min at 6 months and 40–50 min at 12 months. Socio-emotional development was assessed via a maternal questionnaire.

### 2.4. Statistical Analysis

Descriptive statistics and bivariate analyses (Student’s *t*-test, Mann–Whitney U, one-way ANOVA, Kruskal–Wallis, and Pearson correlations) were conducted to explore associations between maternal and infant characteristics and BSID-III scores.

To evaluate developmental trajectories from 6 to 12 months, linear mixed-effects models were applied for each domain. Models included maternal weight status (overweight/obesity) as the main exposure were adjusted for covariates identified in bivariate analyses (maternal IQ, education, parity; infant sex, gestational age, and nBMI. Marginal and conditional R^2^ values were reported to reflect variance explained by fixed and random effects, respectively. Statistical significance was defined as *p* < 0.05.

All statistical analyses were conducted using R version 4.4.1 in RStudio (version 2024.09.1+394, “Cranberry Hibiscus”).

## 3. Results

### 3.1. Maternal and Neonatal Characteristics

Of the 238 mother–infant pairs initially included as part of the OBESO cohort with complete pregnancy follow-up, 127 children did not complete the neurodevelopmental assessments at 6 or 12 months due to the COVID-19 pandemic, and 28 more did not meet other inclusion criteria. Ninety-seven dyads with full neurodevelopmental data at both 6 and 12 months were included in the final analysis.

No significant differences were observed between normal-weight and overweight/obesity groups regarding maternal age, education, or IQ. While not statistically significant, a higher proportion of multiparous women and cesarean deliveries was observed in the overweight/obesity group. Women with overweight/obesity gained significantly less weight during pregnancy (mean ± SD: 6.16 ± 5.32 kg) than those with normal weight (8.36 ± 3.88 kg, *p* = 0.02), and a greater proportion experienced excessive gestational weight gain (27.8% vs. 12.4%, *p* = 0.02). Prevalence of gestational diabetes and preeclampsia was higher in the overweight/obesity group, though not statistically significant. Regarding neonatal outcomes, there were no significant differences between groups in birth weight, gestational age, sex, Apgar scores, or nBMI (Table 1).

### 3.2. Neurodevelopmental Outcomes

In the bivariate analysis, at 6 months of age, neurodevelopmental domain scores did not differ significantly by maternal weight status. However, at 12 months, infants born to mothers with overweight/obesity showed significantly lower language (*p* = 0.002) and socioemotional (*p* = 0.031) scores. Male infants scored lower in cognitive development than females at 6 months, while children of multiparous mothers had reduced socio-emotional scores at both time periods (Table 2).

Neurodevelopmental scores at 6 and 12 months, stratified by infant sex and maternal pre-pregnancy BMI, are presented in Table 3. At 6 months of age, no significant differences were observed across pBMI groups in any neurodevelopmental domain for either sex, although a non-significant trend toward lower cognitive, language, and motor scores was noted among infants born to mothers with overweight/obesity. By 12 months, significant differences emerged. Among females, language scores were lower in the overweight/obesity group (*p* = 0.046). Among males, maternal overweight/obesity was associated with significantly lower scores in cognitive (*p* = 0.039), language (*p* = 0.010), and socioemotional (*p* = 0.029) domains. Motor scores did not differ significantly between groups at either age.

**Figure 1:**
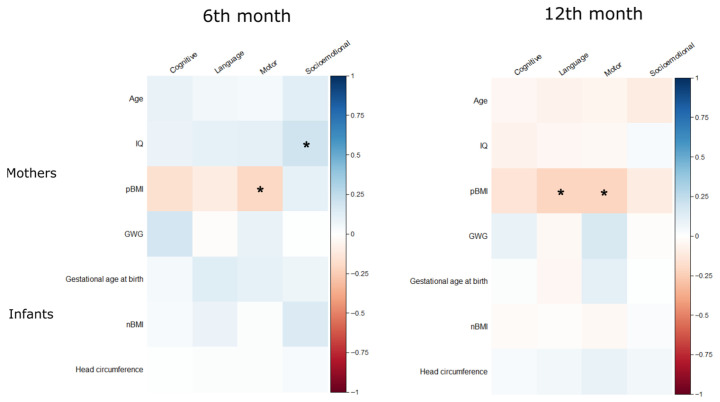
Correlation plots between continuous maternal and neonatal variables and BSID-III scores (cognitive, language, motor, and socioemotional) at 6 and 12 months. Pearson correlation coefficients (* significant values *p* < 0.05). IQ (Intelligence Quotient); pBMI (pregestational Body Mass Index); GWG (Gestational Weight Gain); nBMI (newborn Body Mass Index).

Negative correlations were observed between pBMI and motor scores at 6 months (r = −0.2, *p* = 0.03), and between pBMI and both motor (r = −0.21, *p* = 0.04) and language (r = −0.21, *p* = 0.04) scores at 12 months. Maternal IQ was positively associated with socio-emotional development at 6 months (r = 0.21, *p* = 0.04) (Figure 1).

### 3.3. Developmental Trajectories

**Figure 2:**
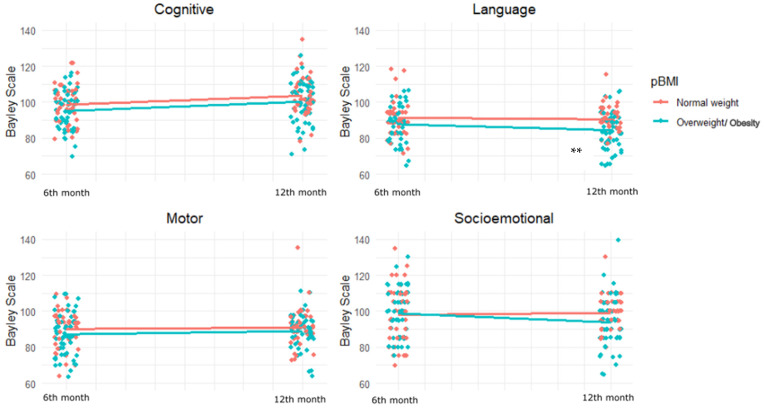
Developmental trajectories at 6 and 12 months for BSID-III comparing infants born to mothers with normal weight vs. overweight/obesity before pregnancy. ** *p* = 0.002.

In mixed-effects models (Figure 2 and Table 4) adjusted for maternal and infant covariates, maternal overweight/obesity was significantly associated with a reduction in language scores from 6 to 12 months (β = −5.44, 95% CI: −8.92 to −1.97, *p* = 0.003). A non-significant trend toward lower cognitive scores was also observed (β = −3.21348, 95% CI: −6.79072 to 0.363772, *p* = 0.07). Additional predictors included female sex (β = 4.05, *p* = 0.027) positively associated with cognitive development, and maternal IQ and nBMI positively associated with socio-emotional scores. Multiparity was negatively associated with socioemotional development (β = −7.06, *p* = 0.003).

These findings indicate that maternal overweight/obesity influences early neurodevelopment, particularly in the language domain, and that both biological and sociodemographic factors contribute to developmental trajectories during the first year of life.

## 4. Discussion

This study explored the association between maternal overweight and obesity before pregnancy with infant neurodevelopmental trajectories during the first year of life. Our findings indicate that infants born to mothers with overweight and obesity exhibited significantly lower language scores, with a downward trend in cognitive development. These effects persisted after adjusting for relevant maternal and neonatal covariates. These results contribute to a growing body of evidence linking maternal metabolic health to early-life brain development.

Several cohort studies have demonstrated long-term cognitive impacts of maternal obesity [34,35,36,37]. The U.S. Collaborative Perinatal Project reported that maternal pregestational obesity was associated with lower full-scale and verbal IQ scores in children at school age [6]. In the PREDO study, children born to mothers with obesity showed lower scores across multiple domains, including communication, motor, problem-solving, and personal-social skills at 3.5 years [38]. The Millennium Cohort Study in the UK also found that maternal pre-pregnancy BMI was negatively associated with children’s cognitive performance at 5 and 7 years of age, even after adjusting for sociodemographic variables [39].

Our findings regarding decreased language scores at 6 and 12 months are consistent with those of the PREOBE study, which reported lower composite and expressive language scores in infants of mothers with obesity. Follow-up analyses showed an initial acceleration in cognitive and language development followed by a decline at 18 months, particularly in the language domain [23]. A recent meta-analysis also highlighted maternal obesity as a significant moderator of poor language outcomes in early childhood [40].

In terms of motor development, negative correlations between maternal pBMI and motor scores at 6 and 12 months in our study echo findings from the Columbia Center for Children’s Environmental Health, which reported that pregestational obesity was associated with lower psychomotor development scores in boys at 3 years of age [41]. The PREOBE study also documented a trend toward lower gross motor scores among infants of mothers with obesity [42].

Emerging evidence also links maternal overweight/obesity to early socio-emotional difficulties. In our cohort, we observed lower socio-emotional scores at 12 months among infants of multiparous and overweight mothers. Similar patterns were noted in the Shanghai Maternal-Child Pairs Cohort (MCPC) and in the PREDO study, which reported impaired emotional and social development in toddlers exposed to maternal obesity [43,44]. These early impairments may reflect precursors of more severe behavioral or neuropsychiatric outcomes later in childhood [45,46].

The biological mechanisms underlying these associations remain under investigation. Maternal obesity has been linked to systemic inflammation, oxidative stress, altered placental signaling, and disruptions in fetal neurogenesis and synaptic maturation [13,47]. Neuroimaging studies reveal cortical thinning in language-related brain regions between neonates born to obese mothers, indicating that increased maternal pBMI has a programming influence on the developing neonate brain functional networks [12,48]. Additionally, differences in infant gut microbiota and breastmilk composition may influence neurodevelopmental trajectories after birth [49].

Given the significant preventive promise of the Developmental Origins of Health and Disease (DOHaD) hypothesis, follow-up studies have been conducted in older children, suggesting that risks of neurodevelopmental impairments (cognitive, language, emotional and IQ) persist throughout early and middle childhood [50,51]. We must highlight the richness of early follow-ups of child development, as we see differences in the scores of the developmental domains that have a significant inverse association with pBMI at the end of the first year of life. Since it is not entirely clear how neurodevelopmental trajectories occur from the infant to early childhood stage, and little is known about the risk factors affecting the downward transition patterns of neurodevelopment [52], our study contributes to the understanding of this gap of knowledge.

Our study also highlighted the role of maternal and neonatal characteristics. Multiparity was negatively associated with socioemotional development, consistent with literature suggesting reduced one-on-one interaction and increased caregiving burden, underscoring the crucial role of maternal factors in early development [53,54,55,56]. Conversely, higher maternal IQ and nBMI were linked to better socioemotional scores, supporting the protective effects of maternal cognitive resources and adequate postnatal growth [57,58,59,60]. However, an unexpected negative correlation was found between maternal education and language scores at 12 months. Educational level and IQ are closely related and recognized as key predictors of child development; nonetheless, the influence is multifactorial and strongly shaped by contextual circumstances [61]. One possible explanation is that mothers with higher education may have less time to dedicate to direct stimulation and interaction with their infants. In line with this, some studies report improvements in certain developmental domains with higher maternal education, while others describe negative effects in behavioral aspects, or even no significant associations at all [59,60]. Although IQ and education are critical predictors, multiple environmental and family factors modulate the development of children’s skills and learning, making this process highly multifactorial and dynamic [61].

Female sex was associated with higher cognitive scores, consistent with previous evidence showing early sex differences in neurodevelopment [62,63,64,65]. A well-documented gender gap in favor of girls during early childhood indicates that sex influences neurodevelopment and mental health in diverse ways. Differences in brain structure and function between males and females are evident throughout development, even prenatally, and are thought to contribute to variations in behavior, cognition, and the risk of neurodevelopmental disorders [64,65].

The strengths of this study include its prospective design, the use of standardized and repeated neurodevelopmental assessments using BSID-III, and adjustment for key maternal and infant covariates. However, several limitations should be acknowledged. First, the relatively small sample size (*n* = 97) may have limited statistical power and increased the risk of type I or II errors. While our findings provide preliminary insights, they should be interpreted with caution and validated in larger cohorts. Second, the study was conducted within a single national context, which may restrict the generalizability of the results. Socioeconomic status, cultural factors, healthcare system characteristics, early nutrition, and early childhood service models could all influence the applicability of these findings in other settings. It is important to note that the women recruited for this study were primarily from middle- and lower-income groups, and this low socioeconomic status may have influenced the outcomes observed. To enhance external validity, future research should include cross-cultural comparisons and recruit larger, more diverse populations.

Another limitation is that pregestational weight was self-reported, which may have introduced misclassification. Moreover, we did not include relevant postnatal exposures —such as parenting style, caregiver mental health, detailed infant feeding practices, or diet and supplement consumption during pregnancy—that are known to influence neurodevelopment. In addition, we relied exclusively on BSID-III scores, whereas previous studies have also documented associations with affective and behavioral outcomes using alternative assessment tools [8,9,17]. Other early-life factors, such as adverse childhood experiences, parenting practices, and caregiver mental health [66,67], must also be considered given their strong influence on developmental trajectories.

Beyond biological mechanisms, maternal obesity may indirectly affect child development through caregiving behaviors. Reduced engagement in physically active interactions—such as outdoor play or movement-based stimulation—could restrict opportunities for motor and cognitive development in infancy. These indirect pathways may contribute to the observed differences in Bayley scores, particularly in motor and language domains. Further research is warranted to investigate caregiving behaviors as potential mediators of the association between maternal obesity and infant neurodevelopment.

Taken together, our findings support the hypothesis of early programming of neurodevelopment through maternal metabolic health. The effects observed in the first year of life, particularly in language and socioemotional domains, warrant close follow-up and early screening that consider other important maternal determinants and environmental factors in child development. Interventions promoting maternal health before and during pregnancy may mitigate risks of early neurodevelopmental delays, with potential long-term benefits for child development and well-being.

## 5. Conclusions

Infants born to mothers with pregestational overweight or obesity showed a decline in certain neurodevelopmental domain scores from 6 to 12 months of age, particularly in language and cognitive trajectories. These findings enhance our understanding of the impact of maternal weight status on early neurodevelopment and highlight the need for closer monitoring and timely interventions during pregnancy and infancy.

## Figures and Tables

**Table 1:** Sociodemographic and clinical data of mother–infant pairs included in the study.

	Normal Weight (*n* = 41)	Overweight/Obesity (*n* = 56)	*p* Value
Mothers			
Age (years)	29.85 ± 4.59	30.42 ± 5.51	0.75
Education level			
Middle and high school	28 (28.9%)	37 (38.1%)	0.26
Higher education	13 (13.4%)	19 (19.6%)	0.29
IQ	91.0 ± 12.0	89.9 ± 10.4	0.65
Parity			
Nulliparous	27 (27.8%)	32 (32.9%)	0.51
Multiparous	14 (14.43%)	24 (24.7%)	0.10
Gestational weight gain (kg)	8.36 ± 3.88	6.16 ± 5.32	0.02
Gestational weight gain (Diagnostic)			
Insufficient	15 (15.4%)	15 (15.4%)	0.99
Adequate	14 (14.4%)	12 (12.4%)	0.69
Excessive	12 (12.4%)	27 (27.8%)	0.02
Gestational diabetes/preeclampsia			
Yes	6 (6.2%)	11 (11.3%)	0.23
No	35 (13.0%)	45 (46.4%)	0.26
Mode of delivery			
Vaginal	22 (22.7%)	27 (27.8%)	0.48
Cesarean section	19 (19.6%)	29 (29.9%)	0.15
Infants			
Gestational age at birth (weeks)	38.72 ± 1.32	38.55 ± 1.92	0.59
Sex			
Male	21 (21.6%)	32 (32.9%)	0.13
Female	20 (20.6%)	24 (24.7%)	0.55
Preterm birth			
Yes	4 (4.1%)	6 (6.2%)	0.52
No	37 (38.1%)	60 (51.5%)	
nBMI	12.410 ± 1.48	12.97 ± 1.56	0.89
Head circumference birth (cm)	33.4 ± 1.39	33.5 ± 1.72	0.55
Birth weight (g)	2900 ± 396	2900 ± 500	0.97
Birth length (cm)	47.30 ± 2.42	47.13 ± 2.79	0.72
Apgar 1 min	7.28 ± 2.06	7.81 ± 1.2	0.14
Apgar 5 min	8.95 ± 0.39	8.94 ± 0.30	0.94

Data are presented in means ± standard deviations or counts and percentage. Pathologies in pregnancy include gestational diabetes and preeclampsia. nBMI: newborn Body Mass Index, IQ: Intelligence Quotient.

**Table 2:** BSID-III scores at 6 and 12 months according to maternal and infant characteristics.

	6th Month				12th Month				Total (97)
	Cognitive	Language	Motor	Socioemotional	Cognitive	Language	Motor	Socioemotional	
Mothers (*n* = 97)									
Education level									
Middle and high school	96.54 ± 9.96	90.00 ± 9.57	88.46 ± 11.24	97.77 ± 14.20	102.46 ± 10.87	88.14 ± 9.55	89.72 ± 9.22	94.94 ± 12.65	65 (67.0%)
Higher education	96.87 ± 11.48	87.97 ± 10.75	87.53 ± 12.38	100.00 ± 15.03	100.31 ± 11.57	82.56 ± 16.29	88.88 ± 12.90	98.44 ± 11.94	32 (33.0%)
*p* value	0.895	0.506	0.677	0.431	0.234	0.097	0.292	0.25	0.356
Parity									
Nulliparous	96.69 ± 10.93	89.49 ± 9.21	87.39 ± 12.34	101.53 ± 13.01	101.10 ± 11.75	85.92 ± 14.64	88.54 ± 12.28	98.05 ± 11.89	38 (39.2%)
Multiparous	96.58 ± 10.3	89.08 ± 11.15	89.34 ± 10.33	93.82 ± 15.44	102.76 ± 10.05	86.89 ± 7.82	90.84 ± 6.87	93.05 ± 12.88	59 (60.8%)
*p* value	0.937	0.584	0.467	0.013	0312	0.888	0.18	0.041	0.459
Pregestational BMI									
Normal weight	98.66 ± 10.25	91.41 ± 9.89	90.17 ± 10.24	98.05 ± 16.08	103.66 ± 10.73	90.56 ± 7.43	91.02 ± 10.71	99.15 ± 9.41	41 (42.3%)
Overweight/obesity	95.18 ± 10.40	87.80 ± 9.82	86.68 ± 12.34	98.84 ± 13.25	100.36 ± 11.24	83.18 ± 14.29	88.29 ± 10.31	93.86 ± 13.96	56 (57.7%)
*p* value	0.135	0.149	0.117	0.791	0.31	0.002	0.396	0.031	0.878
Gestational weight gain									
Weight loss	93.33 ± 13.29	89.67 ± 9.35	84.17 ± 19.02	97.50 ± 13.69	101.67 ± 18.35	87.67 ± 14.72	85.50 ± 13.07	95.83 ± 25.58	6 (6.2%)
Insufficient	94.60 ± 9.89	90.48 ± 10.08	90.60 ± 10.86	95.40 ± 14.36	102.00 ± 10.31	88.72 ± 10.11	90.64 ± 12.23	96.00 ± 9.57	25 (25.8%)
Adequate	97.50 ± 11.07	88.42 ± 11.07	86.65 ± 11.27	102.88 ± 14.01	102.12 ± 11.33	87.27 ± 7.58	87.54 ± 8.56	97.50 ± 12.19	27 (27.8%)
Excessive	97.82 ± 10.12	88.95 ± 10.12	88.05 ± 11.14	97.56 ± 14.86	101.54 ± 10.65	83.69 ± 15.60	90.51 ± 10.35	95.28 ± 12.20	39 (40.2%)
*p* value	0.769	0.673	0.682	0.405	0.830	0.615	0.482	0.977	0.102
GDM/ preeclampsia									
No	95.81 ± 10.75	89.03 ± 10.30	87.68 ± 11.73	98.12 ± 14.35	101.88 ± 11.46	85.74 ± 13.18	89.56 ± 11.31	95.95 ± 13.46	80 (82.5%)
Yes	100.59 ± 7.88	90.76 ± 8.29	90.41 ± 10.89	100.29 ± 15.15	101.18 ± 9.44	88.94 ± 7.28	88.88 ± 5.59	96.76 ± 6.11	17 (17.5%)
*p* value	0.072	0.358	0.466	0.388	0.969	0.393	0.759	0.897	0.78
Route of delivery									
Vaginal	96.84 ± 10.83	89.41 ± 10.10	88.20 ± 11.82	99.49 ± 11.82	101.53 ± 14.08	84.08 ± 10.95	89.45 ± 13.93	95.92 ± 9.07	49 (50.5%)
Cesarean section	96.46 ± 10.10	89.25 ± 9.93	88.10 ± 9.93	97.50 ± 11.44	101.98 ± 14.88	88.56 ± 11.33	89.44 ± 10.23	96.27 ± 11.90	48 (49.5%)
*p* value	0.991	0.902	0.942	0.514	0.658	0.858	0.954	0.954	0.89
Infants (*n* = 97)									
Sex									
Male	95.00 ± 10.38	89.42 ± 8.88	87.32 ± 11.40	99.25 ± 15.11	99.53 ± 11.06	86.43 ± 9.92	90.08 ± 9.12	95.49 ± 13.32	53 (54.6%)
Female	98.64 ± 10.25	89.23 ± 11.23	89.16 ± 11.83	97.61 ± 13.70	104.43 ± 10.63	86.14 ± 14.95	88.68 ± 12.05	96.82 ± 11.47	44 (45.4%)
*p* value	0.049	0.861	0.371	0.633	0.37	0.651	0.263	0.72	0.3
Preterm birth									
No	96.67 ± 10.61	89.61 ± 9.89	88.14 ± 12.04	98.33 ± 14.38	101.78 ± 11.28	86.17 ± 12.75	89.33 ± 10.94	96.09 ± 12.56	87 (89.7%)
Yes	96.50 ± 9.14	86.90 ± 10.84	88.30 ± 6.70	100.00 ± 15.63	101.50 ± 9.73	87.40 ± 8.96	90.40 ± 5.80	96.10 ± 12.24	10 (10.3%)
*p* value	0.962	0.363	0.858	0.711	0.914	0.830	0.525	0.962	0.12

Data are presented in means ± standard deviations for the Bayley-III subscales (cognitive, language, motor, and socioemotional). Pathologies in pregnancy include gestational diabetes and preeclampsia. *p*-values were obtained using Student’s *t*-test, Mann–Whitney U test, one-way ANOVA, or Kruskal–Wallis test, as appropriate. Statistical significance was defined as *p* < 0.05.

**Table 3:** BSID-III scores at 6 and 12 months stratified by infant sex and maternal weight status.

6 Months						
	Female			Male		
	Normal Weight	Overweight/Obesity	*p* Value	Normal Weight	Overweight/Obesity	*p* Value
Cognitive	101.25 ± 10.24	96.46 ± 9.94	0.125	96.19 ± 9.86	94.22 ± 10.78	0.496
Language	92.20 ± 11.99	86.75 ± 10.15	0.116	90.67 ± 7.60	88.59 ± 9.66	0.388
Motor	91.50 ± 9.55	87.21 ± 13.33	0.222	88.90 ± 10.94	86.28 ± 11.75	0.412
Socioemotional	97.00 ± 14.99	98.12 ± 12.84	0.793	99.05 ± 17.37	99.38 ± 13.72	0.942
**12 Months**						
Cognitive	104.00 ± 11.54	104.79 ± 10.05	0.812	103.33 ± 10.17	97.03 ± 11.06	0.039
Language	90.80 ± 8.32	82.25 ± 18.05	0.046	90.33 ± 6.67	83.88 ± 10.92	0.010
Motor	90.85 ± 12.67	86.88 ± 11.46	0.286	91.19 ± 8.77	89.34 ± 9.40	0.470
Socioemotional	98.00 ± 6.57	95.83 ± 14.42	0.515	100.24 ± 11.56	92.38 ± 13.64	0.029

**Table 4:** Results of linear mixed models showing the association of maternal overweight/obesity and other maternal and neonatal characteristics with infant neurodevelopment from 6 to 12 months.

Predictor	β	95% CI		*p* Value	R^2^m	R^2^c
		Lower	Upper			
Cognitive					0.11	0.39
Overweight/obesity	−3.21348	−6.79072	0.363772	0.077681		
Higher education	−0.54558	−4.38046	3.289294	0.778076		
Multiparous	0.718202	−3.02169	4.458094	0.703685		
Maternal IQ	−0.02192	−0.18905	0.145208	0.795001		
Gestational age at birth	0.078311	−1.07223	1.228852	0.892726		
nBMI	0.06884	−1.28041	1.418093	0.91948		
Female	4.046038	0.466961	7.625115	0.027165		
Language					0.1	0.26
Overweight/obesity	−5.44304	−8.91935	−1.96672	0.002505		
Higher education	−3.83191	−7.55858	−0.10523	0.044001		
Multiparous	0.256382	−3.37799	3.890751	0.888843		
Maternal IQ	0.003298	−0.15911	0.165711	0.967905		
Gestational age at birth	0.251222	−0.86686	1.369301	0.656352		
nBMI	0.196762	−1.11442	1.507947	0.766264		
Female	−0.8206	−4.29869	2.657494	0.640362		
Motor					0.05	0.56
Overweight/obesity	−3.16637	−7.17375	0.841017	0.119969		
Higher education	−0.6864	−4.98239	3.609589	0.751628		
Multiparous	2.779343	−1.41024	6.968927	0.190836		
Maternal IQ	0.01883	−0.16839	0.206053	0.842065		
Gestational age at birth	0.957744	−0.33114	2.24663	0.143344		
nBMI	−0.53423	−2.04572	0.977261	0.484332		
Female	−0.53801	−4.54744	3.471425	0.790375		
Socioemotional					0.12	0.4
Overweight/obesity	−1.53999	−5.91128	2.831306	0.485751		
Higher education	2.42417	−2.26194	7.110275	0.30679		
Multiparous	−7.06145	−11.6315	−2.49141	0.002836		
Maternal IQ	0.24423	0.040004	0.448455	0.019637		
Gestational age at birth	−0.36472	−1.77065	1.041205	0.607511		
nBMI	1.704818	0.056069	3.353567	0.042855		
Female	0.387941	−3.98559	4.76147	0.860499		

nBMI = newborn Body Mass Index, IQ = Intelligence Quotient, R^2^m = marginal R^2^, R^2^c = conditional R^2^. Significance set at *p* < 0.05.

## Data Availability

The raw data supporting the conclusions of this article will be made available by the authors without undue reservation. Please contact the corresponding author for data requests.

## Abbreviations

The following abbreviations are used in this manuscript:

OBESO	Cohort Epigenetic and Biochemical Origin of Overweight and Obesity.
pBMI	Pregestational body mass index
IQ	Intelligence Quotient.
PREOBE	Study of maternal nutrition and genetic on the fetal adiposity programming
PREDO	Prediction and prevention of preeclampsia and intrauterine growth restriction study
INPer	National Institute of Perinatology Mexico
WHO	World Health Organization
GWG	Gestational weight gain
BSID-III	Bayley Scales of Infant Development Third Edition
nBMI	Newborn body mass index
Shanghai MCPC	The Shanghai Maternal-Child Pairs Cohort
MRI	Magnetic Resonance Imaging
DOHaD	Developmental Origins of Health and Disease

## References

[1] Obesity and Overweight [Internet]. https://www.who.int/news-room/fact-sheets/detail/obesity-and-overweight.

[2] Campos-Nonato I., Galván-Valencia Ó., Hernández-Barrera L., Oviedo-Solís C., Barquera S. (2023). Prevalence of obesity and associated risk factors in Mexican adults: Results of the Ensanut 2022. Salud Publica Mex..

[3] Bolton J.L., Bilbo S.D. (2014). Developmental programming of brain and behavior by perinatal diet: Focus on inflammatory mechanisms. Dialogues Clin. Neurosci..

[4] Godfrey K.M., Reynolds R.M., Prescott S.L., Nyirenda M., Jaddoe V.W.V., Eriksson J.G., Broekman B.F.P. (2017). Influence of maternal obesity on the long-term health of offspring. Lancet Diabetes Endocrinol..

[5] Hao X., Lu J., Yan S., Tao F., Huang K. (2022). Maternal Pre-Pregnancy Body Mass Index, Gestational Weight Gain and Children’s Cognitive Development: A Birth Cohort Study. Nutrients.

[6] Huang L., Yu X., Keim S., Li L., Zhang L., Zhang J. (2014). Maternal prepregnancy obesity and child neurodevelopment in the Collaborative Perinatal Project. Int. J. Epidemiol..

[7] Tong L., Kalish B.T. (2021). The impact of maternal obesity on childhood neurodevelopment. J. Perinatol..

[8] Edlow A.G. (2017). Maternal obesity and neurodevelopmental and psychiatric disorders in offspring. Prenat. Diagn..

[9] Sanchez C.E., Barry C., Sabhlok A., Russell K., Majors A., Kollins S.H., Fuemmeler B.F. (2018). Maternal pre-pregnancy obesity and child neurodevelopmental outcomes: A meta-analysis. Obes. Rev..

[10] Adane A.A., Mishra G.D., Tooth L.R. (2016). Maternal pre-pregnancy obesity and childhood physical and cognitive development of children: A systematic review. Int. J. Obes..

[11] Gage S.H., Lawlor D.A., Tilling K., Fraser A. (2013). Associations of maternal weight gain in pregnancy with offspring cognition in childhood and adolescence: Findings from the Avon Longitudinal Study of Parents and Children. Am. J. Epidemiol..

[12] Rajasilta O., Häkkinen S., Björnsdotter M., Scheinin N.M., Lehtola S.J., Saunavaara J., Parkkola R., Lähdesmäki T., Karlsson L., Karlsson H. (2021). Maternal pre-pregnancy BMI associates with neonate local and distal functional connectivity of the left superior frontal gyrus. Sci. Rep..

[13] Nazzari S., Frigerio A. (2020). The programming role of maternal antenatal inflammation on infants’ early neurodevelopment: A review of human studies: Special Section on “Translational and Neuroscience Studies in Affective Disorders” Section Editor, Maria Nobile MD, PhD. J. Affect. Disord..

[14] van der Burg J.W., Sen S., Chomitz V.R., Seidell J.C., Leviton A., Dammann O. (2016). The role of systemic inflammation linking maternal BMI to neurodevelopment in children. Pediatr. Res..

[15] Agarwal S., Scher M.S. (2022). Fetal-neonatal neurology program development: Continuum of care during the first 1000 days. J. Perinatol..

[16] Hadders-Algra M. (2021). Early Diagnostics and Early Intervention in Neurodevelopmental Disorders-Age-Dependent Challenges and Opportunities. J. Clin. Med. Res..

[17] Han V.X., Patel S., Jones H.F., Nielsen T.C., Mohammad S.S., Hofer M.J., Gold W., Brilot F., Lain S.J., Nassar N. (2021). Maternal acute and chronic inflammation in pregnancy is associated with common neurodevelopmental disorders: A systematic review. Transl. Psychiatry..

[18] Darling J.C., Bamidis P.D., Burberry J., Rudolf M.C.J. (2020). The First Thousand Days: Early, integrated and evidence-based approaches to improving child health: Coming to a population near you?. Arch. Dis. Child..

[19] Scher M.S. (2021). “The First Thousand Days” Define a Fetal/Neonatal Neurology Program. Front. Pediatr..

[20] Zapata-Tarrés M.M. (2025). The first 1000 days of life: The great opportunity. Bol. Med. Hosp. Infant. Mex..

[21] Draper C.E., Yousafzai A.K., McCoy D.C., Cuartas J., Obradović J., Bhopal S., Fisher J., Jeong J., Klingberg S., Milner K. (2024). The next 1000 days: Building on early investments for the health and development of young children. Lancet.

[22] Dan B. (2025). The first 1000 days: A critical window for neurodevelopmental trajectories and interventions—And for parents. Dev. Med. Child. Neurol..

[23] Torres-Espinola F.J., Berglund S.K., García-Valdés L.M., Segura M.T., Jerez A., Campos D., Moreno-Torres R., Rueda R., Catena A., Pérez-García M. (2015). Maternal Obesity, Overweight and Gestational Diabetes Affect the Offspring Neurodevelopment at 6 and 18 Months of Age—A Follow Up from the PREOBE Cohort. PLoS ONE.

[24] Girchenko P., Lahti M., Tuovinen S., Savolainen K., Lahti J., Binder E.B., Reynolds R.M., Entringer S., Buss C., Wadhwa P.D. (2017). Cohort Profile: Prediction and prevention of preeclampsia and intrauterine growth restriction (PREDO) study. Int. J. Epidemiol..

[25] Kato T., Nishimura T., Takahashi N., Harada T., Okumura A., Iwabuchi T., Nomura Y., Senju A., Tsuchiya K.J., Takei N. (2022). Identification of neurodevelopmental transition patterns from infancy to early childhood and risk factors predicting descending transition. Sci. Rep..

[26] Lohman T.J., Roache A.F., Martorell R. (1988). Anthropometric Standardization Reference Manual.

[27] World Health Organization (2003). Diet, Nutrition, and the Prevention of Chronic Diseases: Report of a Joint WHO/FAO Expert Consultation.

[28] Wechsler D. (2011). Wechsler Abbreviated Scale of Intelligence—Second Edition (WASI II).

[29] Institute of Medicine, National Research Council (2009). Weight Gain During Pregnancy: Reexamining the Guidelines.

[30] World Health Organization (2013). Diagnostic Criteria and Classification of Hyperglycaemia First Detected in Pregnancy [Internet].

[31] World Health Organization (2011). WHO Recommendations for Prevention and Treatment of Pre-Eclampsia and Eclampsia [Internet].

[32] World Health Organization (2018). Preterm Birth [Internet].

[33] Bayley N. (2005). Bayley Scales of Infant and Toddler Development.

[34] Dong X., Zhou A. (2023). Associations of maternal pre-pregnancy body mass index and gestational weight gain with risk of offspring neurodevelopment at 2 years: A Chinese birth cohort study. Front. Pediatr..

[35] Duffany K.O., McVeigh K.H., Kershaw T.S., Lipkind H.S., Ickovics J.R. (2016). Maternal Obesity: Risks for Developmental Delays in Early Childhood. Matern. Child. Health J..

[36] Heikura U., Taanila A., Hartikainen A.L., Olsen P., Linna S.L., von Wendt L., Järvelin M.R. (2008). Variations in prenatal sociodemographic factors associated with intellectual disability: A study of the 20-year interval between two birth cohorts in northern Finland. Am. J. Epidemiol..

[37] Babaei M., Machle C.J., Mokhtari P., Ottino González J., Schmidt K.A., Alderete T.L., Adise S., Peterson B.S., Goran M.I. (2024). Pre-pregnancy maternal obesity and infant neurodevelopmental outcomes in Latino infants. Obesity.

[38] Girchenko P., Tuovinen S., Lahti-Pulkkinen M., Lahti J., Savolainen K., Heinonen K., Pyhälä R., Reynolds R.M., Hämäläinen E., Villa P.M. (2018). Maternal early pregnancy obesity and related pregnancy and pre-pregnancy disorders: Associations with child developmental milestones in the prospective PREDO Study. Int. J. Obes..

[39] Basatemur E., Gardiner J., Williams C., Melhuish E., Barnes J., Sutcliffe A. (2013). Maternal prepregnancy BMI and child cognition: A longitudinal cohort study. Pediatrics.

[40] Arabiat D., Al Jabery M., Jenkins M., Kemp V., Whitehead L., Adams G. (2021). Language abilities in children born to mothers diagnosed with diabetes: A systematic review and meta-analysis. Early Hum. Dev..

[41] Nichols A.R., Rundle A.G., Factor-Litvak P., Insel B.J., Hoepner L., Rauh V., Perera F., Widen E.M. (2020). Prepregnancy obesity is associated with lower psychomotor development scores in boys at age 3 in a low-income, minority birth cohort. J. Dev. Orig. Health Dis..

[42] Berglund S.K., Torres-Espínola F.J., García-Valdés L., Segura M.T., Martínez-Zaldívar C., Padilla C., Rueda R., García M.P., McArdle H.J., Campoy C. (2017). The impacts of maternal iron deficiency and being overweight during pregnancy on neurodevelopment of the offspring. Br. J. Nutr..

[43] Zhang S., Ma X., Wei Q., Zhang Y., Wang L., Shi H. (2022). Maternal Pre-Pregnancy BMI and Gestational Weight Gain Modified the Association between Prenatal Depressive Symptoms and Toddler’s Emotional and Behavioral Problems: A Prospective Cohort Study. Nutrients.

[44] Girchenko P., Lahti-Pulkkinen M., Lahti J., Pesonen A.-K., Hämäläinen E., Villa P.M., Kajantie E., Laivuori H., Reynolds R.M., Räikkönen K. (2018). Neonatal regulatory behavior problems are predicted by maternal early pregnancy overweight and obesity: Findings from the prospective PREDO Study. Pediatr. Res..

[45] Mina T.H., Lahti M., Drake A.J., Denison F.C., Räikkönen K., E Norman J., Reynolds R.M. (2017). Prenatal exposure to maternal very severe obesity is associated with impaired neurodevelopment and executive functioning in children. Pediatr. Res..

[46] Robinson S.L., Ghassabian A., Sundaram R., Trinh M.-H., Lin T.-C., Bell E.M., Yeung E. (2020). Parental Weight Status and Offspring Behavioral Problems and Psychiatric Symptoms. J. Pediatr..

[47] Neri C., Edlow A.G. (2015). Effects of Maternal Obesity on Fetal Programming: Molecular Approaches. Cold Spring Harb. Perspect. Med..

[48] Na X., Phelan N.E., Tadros M.R., Wu Z., Andres A., Badger T.M., Glasier C.M., Ramakrishnaiah R.R., Rowell A.C., Wang L. (2021). Maternal Obesity during Pregnancy is Associated with Lower Cortical Thickness in the Neonate Brain. AJNR Am. J. Neuroradiol..

[49] Ionescu M.I., Zahiu C.D.M., Vlad A., Galos F., Gradisteanu Pircalabioru G., Zagrean A.M., O’Mahony S.M. (2025). Nurturing development: How a mother’s nutrition shapes offspring’s brain through the gut. Nutr. Neurosci..

[50] Widen E.M., Kahn L.G., Cirillo P., Cohn B., Kezios K.L., Factor-Litvak P. (2018). Prepregnancy overweight and obesity are associated with impaired child neurodevelopment. Matern. Child. Nutr..

[51] Krzeczkowski J.E., Lau A., Fitzpatrick J., Tamana S., Smithson L., de Souza R., Kozyrskyj A.L., Lefebvre D., Becker A.B., The CHILD Study Investigators (2019). Maternal Metabolic Complications in Pregnancy and Offspring Behavior Problems at 2 Years of Age. Matern. Child. Health J..

[52] Costa Wiltgen A., Valentini N.C., Beltram Marcelino T., Santos Pinto Guimarães L., Homrich Da Silva C., Rombaldi Bernardi J., Goldani M.Z. (2023). Different intrauterine environments and children motor development in the first 6 months of life: A prospective longitudinal cohort. Sci. Rep..

[53] Islam M.M., Khan M.N. (2023). Early childhood development and its association with maternal parity. Child. Care Health Dev..

[54] Glynn L.M. (2012). Increasing parity is associated with cumulative effects on memory. J. Women’s Health.

[55] Hsu Y.C., Chen C.T., Yang H.J., Chou P. (2019). Family structure, birth order, and aggressive behaviors among school-aged boys with attention deficit hyperactivity disorder (ADHD). Soc. Psychiatry Psychiatr. Epidemiol..

[56] Reimelt C., Wolff N., Hölling H., Mogwitz S., Ehrlich S., Martini J., Roessner V. (2021). Siblings and Birth Order-Are They Important for the Occurrence of ADHD?. J. Atten. Disord..

[57] Sajewicz-Radtke U., Łada-Maśko A., Olech M., Jurek P., Bieleninik Ł., Radtke B.M. (2025). Association between parental education level and intelligence quotient of children referred to the mental healthcare system: A cross-sectional study in Poland. Sci. Rep..

[58] Lean R.E., Paul R.A., Smyser C.D., Rogers C.E. (2018). Maternal intelligence quotient (IQ) predicts IQ and language in very preterm children at age 5 years. J. Child. Psychol. Psychiatry.

[59] Harding J.F. (2015). Increases in maternal education and low-income children’s cognitive and behavioral outcomes. Dev. Psychol..

[60] Zhao Y.K., Li M., Shi T.T., Feng M.M., Hu L.L. (2024). Association of premature birth and maternal education level on attention deficit hyperactivity disorder in children: A meta-analysis. World J. Psychiatry.

[61] Jackson M., Kiernan K., McLanahan S. (2017). Maternal Education, Changing Family Circumstances, and Children’s Skill Development in the United States and UK. Ann. Am. Acad. Pol. Soc. Sci..

[62] Bando R., Lopez-Boo F., Fernald L., Gertler P., Reynolds S. (2024). Gender Differences in Early Child Development: Evidence from Large-Scale Studies of Very Young Children in Nine Countries. J. Econ. Race Policy.

[63] Brandlistuen R.E., Flatø M., Stoltenberg C., Helland S.S., Wang M.V. (2021). Gender gaps in preschool age: A study of behavior, neurodevelopment and pre-academic skills. Scand. J. Public Health.

[64] Rice L.C., Rochowiak R.N., Plotkin M.R., Rosch K.S., Mostofsky S.H., Crocetti D. (2024). Sex Differences and Behavioral Associations with Typically Developing Pediatric Regional Cerebellar Gray Matter Volume. Cerebellum.

[65] Cook K.M., De Asis-Cruz J., Lopez C., Quistorff J., Kapse K., Andersen N., Vezina G., Limperopoulos C. (2022). Robust sex differences in functional brain connectivity are present in utero. Cereb. Cortex.

[66] Short A.K., Baram T.Z. (2019). Early-life adversity and neurological disease: Age-old questions and novel answers. Nat. Rev. Neurol..

[67] Bhutta Z.A., Bhavnani S., Betancourt T.S., Tomlinson M., Patel V. (2023). Adverse childhood experiences and lifelong health. Nat. Med..

